# Macroscopic changes during negative pressure wound therapy of the open abdomen using conventional negative pressure wound therapy and NPWT with a protective disc over the intestines

**DOI:** 10.1186/1471-2482-11-10

**Published:** 2011-04-29

**Authors:** Sandra Lindstedt, Malin Malmsjö, Johan Hansson, Joanna Hlebowicz, Richard Ingemansson

**Affiliations:** 1 Department of Cardiothoracic Surgery, Lund University and Skåne University Hospital, Lund, Sweden; 2 Department of Ophthalmology, Lund University and Skåne University Hospital, Lund, Sweden; 3 Institution of Surgical Sciences, Faculty of Medicine, Uppsala University, Uppsala, Sweden; 4 Department of Medicine, Lund University and Skåne University Hospital, Lund, Sweden

**Keywords:** negative pressure wound therapy, open abdomen, macroscopic changes, intestinal wall

## Abstract

**Background:**

Higher closure rates of the open abdomen have been reported with negative pressure wound therapy (NPWT) than with other wound management techniques. However, the method has occasionally been associated with increased development of fistulae. We have previously shown that NPWT induces ischemia in the underlying small intestines close to the vacuum source, and that a protective disc placed between the intestines and the vacuum source prevents the induction of ischemia. In the present study we compare macroscopic changes after 12, 24, and 48 hours, using conventional NPWT and NPWT with a protective disc between the intestines and the vacuum source.

**Methods:**

Twelve pigs underwent midline incision. Six animals underwent conventional NPWT, while the other six pigs underwent NPWT with a protective disc inserted between the intestines and the vacuum source. Macroscopic changes were photographed and quantified after 12, 24, and 48 hours of NPWT.

**Results:**

The surface of the small intestines was red and mottled as a result of petechial bleeding in the intestinal wall in all cases. After 12, 24 and 48 hours of NPWT, the area of petechial bleeding was significantly larger when using conventional NPWT than when using NPWT with the protective disc (9.7 ± 1.0 cm^2 ^vs. 1.8 ± 0.2 cm^2^, p < 0.001, 12 hours), (14.5 ± 0.9 cm^2 ^vs. 2.0 ± 0.2 cm^2^, 24 hours) (17.0 ± 0.7 cm^2 ^vs. 2.5 ± 0.2 cm^2 ^with the disc, p < 0.001, 48 hours)

**Conclusions:**

The areas of petechial bleeding in the small intestinal wall were significantly larger following conventional NPWT after 12, 24 and 48 hours, than using NPWT with a protective disc between the intestines and the vacuum source. The protective disc protects the intestines, reducing the amount of petechial bleeding.

## Background

An injury to the abdomen or abdominal surgery can result in a wound that cannot be closed immediately. It may be necessary to leave the wound open to allow further treatment, or to allow infection to clear. In this type of open abdomen, laparostomy, the internal organs, including the bowel, may be exposed. Treatment of laparostomy with negative pressure wound therapy (NPWT) for abdominal sepsis and abdominal compartment syndrome has resulted in a high rate of successful abdominal closure. In addition, patients recover more rapidly. In patients with abdominal compartment syndrome, decompressed laparotomy with temporary closure with NPWT might be crucial, whereas in abdominal sepsis and peritonitis NPWT's draining effect and reduction of bacterial load is thought to be of great importance [1-3]. The primary goals of NWPT of the open abdomen include the avoidance of mechanical contamination of abdominal viscera, active removal of exudates, third space fluid loss estimation, and infection control [4]. In this procedure a permeable film is placed over the abdominal contents, and a foam sponge, or other porous dressing, is placed on top. A drainage tube is inserted into the porous dressing. The entire area and surrounding skin is then sealed with drapes. Negative pressure, often between 125 and 150 mmHg, is applied by a vacuum pump, which removes excess blood and fluid from around the bowel. The main purpose of this treatment is to remove contaminated effluent from the peritoneal cavity. The use of airtight dressings and NPWT in managing the open abdomen has improved care and increased the possibility of closure of the open abdomen. However, the method has occasionally been associated with increased development of intestinal and enteroatmospheric fistulae [5-9].

We have previously shown that NPWT induces ischemia in the wall of the small intestine [10]. We have also shown that a protective disc, placed between the intestines and the vacuum source, protects the intestines from ischemia [10]. Persistent ischemia in the intestinal wall may explain why conventional NWPT has been associated with development of fistulae. In the present study, we examine the macroscopic changes after 12, 24, and 48 hours of conventional NPWT and NPWT with a protective disc between the intestines and the vacuum source. To the best of our knowledge, no such study has previously been conducted.

## Material and method

### Experimental animals

Twelve domestic pigs of both sexes, with a median weight of 60 kg, were used. The animals were fasted overnight but given free access to water. The investigation complied with the "Guide for the Care and Use of Laboratory Animals", as recommended by the U.S. National Institutes of Health, and published by the National Academies Press (1996), and to local legislation. The study design was approved by the ethical committee on animal experiments in Region Skane, Sweden.

### Anesthesia

All the animals were pre-medicated intramuscularly with ketamine (30 mg/kg) before being brought into the laboratory. Before commencing surgery, sodium thiopental (5 mg/kg), atropine (0.02 mg/kg) and pancuronium (0.5 mg/kg) were given intravenously. Intubation was performed with a Portex endotracheal tube (7.5 mm internal diameter, Medcompare, South San Francisco, CA). A servo-ventilator (Siemens Elema 300A, Stockholm, Sweden) was used for mechanical ventilation throughout the experiments. The ventilator settings used were: minute volume = 100 ml/kg, FiO2 = 0.5, breathing frequency = 16 breaths/minute and positive end expiratory pressure = 5 cmH2O. Anesthesia and muscular paralysis were maintained by continuous intravenous infusion of 8-10 mg/kg/hour propofol (Diprivan, AstraZeneca, Sweden), 0.15 mg/kg/hour fentanyl (Leptanal, Lilly, France) and 0.6 mg/kg/hour pancuronium (Pavulon, Organon Teknika, Boxtel, the Netherlands).

### Surgical procedure

A 30 cm long midline incision was made. The V.A.C.^® ^Granu Foam™ Abdominal Dressing System (KCI, San Antonio, TX, USA), was used. The visceral protective layer was cut to the appropriate size, extending into the paracolic gutters on both sides (about 30 cm wide and 35 cm long). A layer of polyurethane foam (V.A.C.^® ^Granu Foam™) was placed on top of the visceral protective layer between the wound edges. The wound was covered with self-adhesive polyethylene drape, and a track pad was inserted through the drape (all from KCI, San Antonio, TX, USA) and connected to a continuous vacuum source. Heart frequency and ventilator parameters were recorded throughout the experiments.

### Experimental protocol

**Figure 1:**
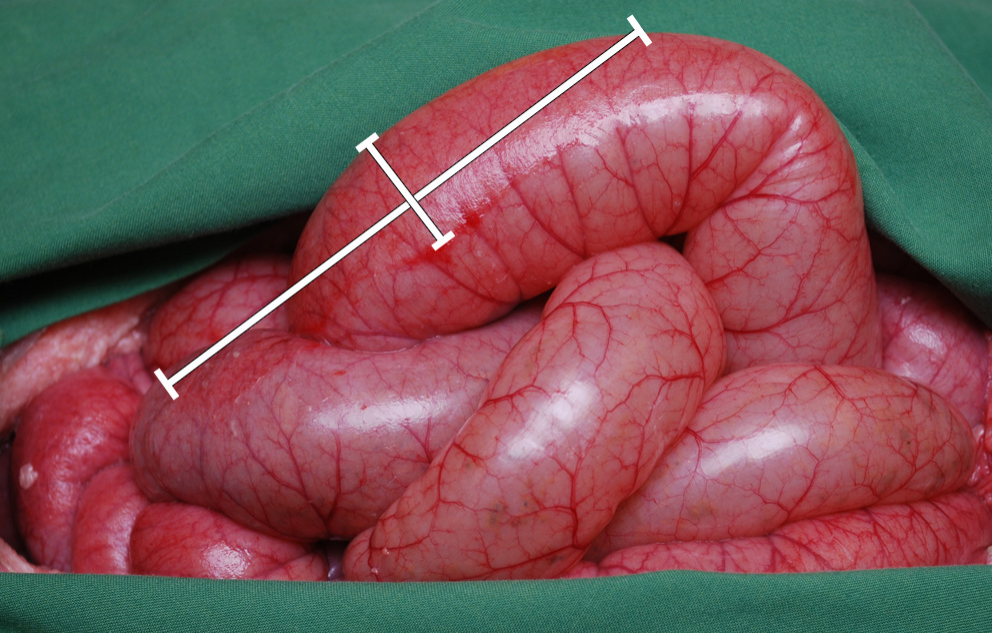
**Photograph of the intestines in a porcine open abdominal wound after conventional NPWT at -120 mmHg in the absence of a protective disc between the intestines and the vacuum source**. Red, mottled areas can be seen due to intestinal petechial bleeding. The area of bleeding was determined by measuring the length and width.

The pigs were divided into two groups of six animals. In one group, a rigid protective disc was inserted between the intestines and the vacuum source before the application of NPWT, while the other group was exposed to NPWT without the disc (conventional NPWT). The animals were treated with a continuous negative pressure of -120 mmHg for 48 hours. The NPWT dressing was changed after 12, and 24 hours. The intestines were inspected with regard to injury after 12, 24, and 48 hours. The length and width of the area affected by petechial bleeding on the surface of the intestinal walls were measured and the area was calculated (Figure 1).

### The protective disc

The protective disc placed between the dressing and the intestines was made out of bio-compatible plastic that could withstand the force of a negative pressure of -50 mmHg. The disc was 60 × 60 cm and was then cut to appropriate size. The disc had multiple small perforations all over the disc area. The disc was flexible and approximately 3 mm thick.

### Calculations and statistics

Calculations and statistical analysis were performed using GraphPad 5.0 software (San Diego, CA, USA). Statistical analysis was performed using the Mann-Whitney test when comparing two groups, and the Kruskal-Wallis test with Dunn's test for multiple comparisons when comparing three groups or more. Significance was defined as *p *< 0.05 (*), *p *< 0.01 (**), *p *< 0.001 (***) and *p *> 0.05 (not significant, n.s.). Values are shown as means and SEM.

## Results

**Figure 2:**
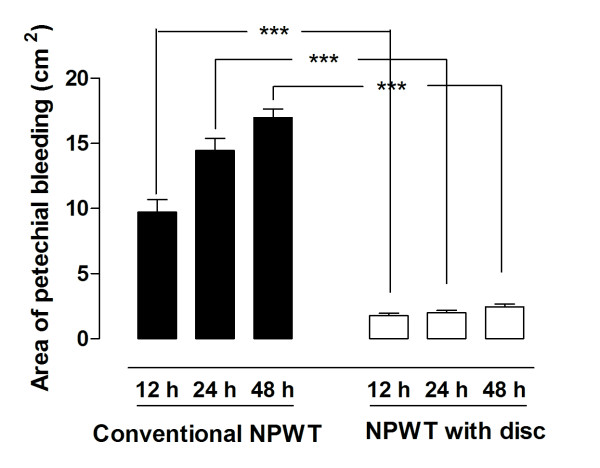
**The area of petechial bleeding following NPWT at -120 mmHg after 12, 24, and 48 hours, with conventional NPWT and NPWT with a protective disc inserted between the intestines and the vacuum source**. Results are presented as the mean of 6 values ± SEM. Statistical analysis was performed using the Mann-Whitney test. Significance was defined as p < 0.05 (*), p < 0.01 (**), p < 0.001 (***) and p > 0.05 (not significant, n.s.). The area of petechial bleeding was smaller when a protective disc was used during NPWT.

**Figure 3:**
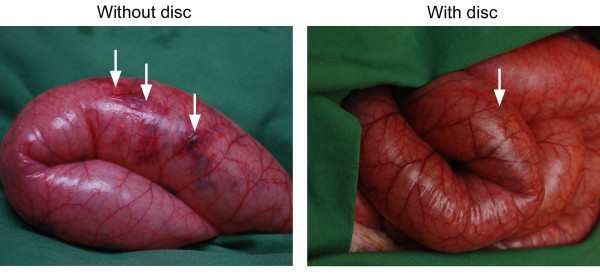
**Photographs of the intestines in a porcine open abdominal wound treated with conventional NPWT (left) and NPWT with a protective disc between the intestines and the vacuum source (right) after 48 hours**. It can clearly be seen that the areas of red, mottled intestines due to petechial bleeding are smaller after NPWT with the protective disc (right).

The surface of the small intestines was red and mottled as a result of petechial bleeding in the wall of the small intestinal in all cases following NPWT (Figure 1). After 12 hours of NPWT, the area of petechial bleeding was significantly larger when using conventional NPWT than when using NPWT with the protective disc (9.7 ± 1.0 cm^2 ^vs. 1.8 ± 0.2 cm^2^, p < 0.001, Figures 2 &3). The area of petechial bleeding was only slightly larger after 24 hours of NPWT than after 12 hours (14.5 ± 0.9 cm^2 ^following conventional NPWT, and 2.0 ± 0.2 cm^2^, following NPWT with the disc, p < 0.001, Figures 2 &3). The area of petechial bleeding was only slightly larger after 48 hours of NPWT than after 12 and 24 hours (17.0 ± 0.7 cm^2 ^with conventional NPWT and 2.5 ± 0.2 cm^2 ^with the disc, p < 0.001, Figures 2 &3).

## Discussion

Managing patients with an open abdomen is not a new problem; however it is still challenging, even for the most experienced clinicians. Life-sustaining emergency surgery on patients with severe abdominal injuries is often accompanied by visceral edema, retroperitoneal hematoma or packing of the abdominal cavity. This is also the case in re-laparotomies carried out to access intestinal viability or to control secondary bleeding after damage control laparotomies, or in connection with intra-abdominal infections. The pressure of forced abdominal wall closure, or an abdominal infection, may lead to ischemia and necrosis of the intestines and the abdominal fascia, the latter resulting in abdominal rupture with subsequent development of an abdominal wall hernia [1-3,11,12]. Laparotomies associated with damage control including packing, the occurrence of abdominal compartment syndrome or severe septic intra-abdominal complications require repeated revisions of the abdominal cavity. All these procedures result in an open abdomen, which does not permit primary closure of the fascia and requires temporary abdominal closure. If the abdomen is not closed in the early postoperative period, the combination of adhesions and fascia retraction frequently makes primary fascia closure impossible, and a planned ventral hernia is often required [1-3,11,12].

Abdominal NPWT differs from the NPWT of other wounds in the application of a perforated, thin polyethylene film between the viscera and the anterior abdominal wall. This prevents the adherence of the viscera to the peritoneum, and allows the abdominal wall to slide over the loops of the bowel. At the same time, NPWT has a drainage effect, facilitating the reduction of peritoneal fluid and bacteria [13,14]. Although higher closure rates of the abdomen have been reported with NWPT compared with other techniques, complications such as fistulae, intra-abdominal abscesses, and wound dehiscence have occasionally been reported during NPWT of the open abdomen [13,14].

We have previously shown that conventional NWPT induces ischemia in the intestinal wall close to the dressing, and that the degree of ischemia is related to the amount of pressure applied [10]. We have also shown that a protective disc placed between the intestines and the vacuum source prevents ischemia [10]. Persistent ischemia in the intestinal wall may explain the development of fistulae with conventional NWPT, although the underlying causes are not fully understood. One mechanism that might cause ischemia is the suction force, since the degree of ischemia increases with the amount of negative pressure applied. Another mechanism that might induce ischemia in the small intestinal wall during conventional NPWT, is the deformation and hernia of the underlying tissue, i.e. small intestines bulging into the space between the wound edges. NWPT with a disc between the intestines and the vacuum source prevents the intestines from bulging into the space between the wound edges, and this could explain why ischemia is prevented. Similar problems following conventional NPWT have been seen in cardiac surgery. A lethal complication following NPWT for post-operative deep sternal wound infection is right ventricle rupture, the incidence being 4 to 7% [15,16]. We have previously described the cause of heart rupture in pigs using magnetic resonance imaging [15,16]. The heart was shown to be drawn up towards the thoracic wall, the right ventricle bulged into the space between the sternal edges, and the sharp edges of the sternum protruded into the anterior surface of the heart [15]. These events could be prevented by inserting a rigid disc between the anterior part of the heart and the inside of the thoracic wall [15].

In the present study, we compared the macroscopic changes after 12, 24, and 48 hours of conventional NPWT and NPWT with a protective disc between the intestines and the vacuum source. The surface of the small intestines showed petechial bleeding in the intestinal wall in all cases. However, the area of petechial bleeding in the small intestinal wall was significantly smaller when using a protective disc between the intestines and the vacuum source, after 12, 24, and 48 hours. We used a pressure of -120 mmHg since this is the level most often used clinically. We have previously shown that NPWT of the open abdomen induce a decrease in microvascular blood flow in the intestinal loops close to the dressing and that a protective disc over the intestines restored the blood flow [10]. Based on those data we believe that the reason for petechial bleeding is secondary to ischemia.

We used healthy pigs with healthy intestines without any signs of ischemic areas or trauma. In the clinical situation infection or trauma may be present, and the results obtained in this study may not reflect those that can be expected in the clinical situation. Further studies, for example, on the induction of ischemia in the mesenteric arteries, may be of interest as intestines that have been exposed to trauma, infection, or ischemia could be expected to be more fragile than healthy intestines, and may suffer greater damage after exposure to conventional NPWT. Most probably different levels of NPWT induce different degrees of macroscopic changes. In this study we choose -120 mmHg since it's a pressure level that often is used clinically. Macroscopic changes are presumable less at for example a negative pressure of -75 mmHg. It might, however inflict on the draining effect.

## Conclusion

The surface of the small intestines showed petechial bleeding in the intestinal wall in all cases following NPWT. Conventional NPWT showed significantly larger areas of petechial bleeding in the small intestinal wall after 12, 24, and 48 hours, than using NPWT with a protective disc between the intestines and the vacuum source. The protective disc protects the intestines, reducing the amount of petechial bleeding. Damage such as petechial bleeding may promote the development of fistulae.

## Competing interests

The authors declare that they have no competing interests.

## Authors' contributions

SL, RI & MM carried out the experimental studies. SL drafted the manuscript. JH & JH participated in the sequence alignment. SL, JH, RI participated in the design of the study and performed the statistical analysis. All authors read and approved the final manuscript.

## Pre-publication history

The pre-publication history for this paper can be accessed here:

http://www.biomedcentral.com/1471-2482/11/10/prepub
